# Genetically proxied glucagon-like peptide-1 receptor perturbation and risk of mood disorders: a Mendelian randomization study

**DOI:** 10.1186/s12888-025-07152-0

**Published:** 2025-08-06

**Authors:** Yaejin Jeon, Ju Han Kim

**Affiliations:** Seoul National University Biomedical Informatics (SNUBI), Department of Biomedical Sciences, Seoul National University College of Medicine, Seoul, 03080 Republic of Korea

**Keywords:** GLP1R, Glycemic control, Bipolar disorder, Major depressive disorder, Mendelian randomization

## Abstract

**Background:**

Glucagon-like peptide-1 receptor (GLP1R) agonists have gained attention for their role in diabetes treatment along with their diverse effects, such as appetite suppression, suggesting potential psychiatric benefits. This study aimed to assess the effect of GLP1R perturbation on mood disorders based on protein and biomarker levels using Mendelian randomization (MR) approach.

**Methods:**

We conducted two-sample MR using summary statistics for GLP1R plasma levels (*n* = 3,301) from the INTERVAL study, glycated hemoglobin (HbA1c) levels (*n* = 128,610) from the Meta-Analyses of Glucose and Insulin-related traits Consortium, and bipolar disorder (BD: 371 cases/360,823 controls) and major depressive disorder (MDD: 776 cases/360,418 controls) incidences from the UK Biobank. Genetic variants associated with the plasma levels of GLP1R and HbA1c were used as proxies for the variation in GLP1R.

**Results:**

GLP1R level was significantly associated with a reduced risk of MDD (odds ratio [OR] = 0·9988, 95% confidence interval [CI] = 0·9978-0·9999, *P* = 0·0291) and of BD (OR = 0·9990, 95% CI = 0·9982-0·9998, *P* = 0·0182). GLP1R’s HbA1c level-lowering effect was significantly associated with a decreased risk of BD (OR = 0·9786, 95% CI = 0·9613-0·9962, *P* = 0·0175) but not with MDD.

**Conclusions:**

GLP1R perturbation may have protective effects on MDD and BD through different mechanisms, although additional clinical trials are required to determine the therapeutic implications.

**Trial registration:**

Clinical trial number not applicable.

**Supplementary Information:**

The online version contains supplementary material available at 10.1186/s12888-025-07152-0.

## Background

Glucagon-like peptide-1 receptor (GLP1R) agonists, GLP1 analogues that are resistant to degradation by dipeptidyl peptidase 4, are used to treat type 2 diabetes mellitus (T2DM) [1]. GLP1R agonists help lower blood sugar levels by stimulating the release of insulin and suppressing glucagon secretion [2]. Beyond their metabolic effects, recent studies have reported antidepressant effects of GLP1R agonists. Clinical trials have reported improvements in depression rating scales among GLP1R agonist users [3]. However, the effects of GLP1R agonists on the incidence of major depressive disorder (MDD) have shown mixed results in a systematic review [4]. Two epidemiological studies suggested decreased risks of MDD among GLP1R agonist users, whereas two other studies suggested no effect [5–8]. For bipolar disorder (BD), evidence from human clinical trials remains limited despite its clinical significance [9, 10]. However, a pilot study suggested that liraglutide altered frontal and striatal volumes and improved cognitive function in patients with BD [11]. 

Therefore, human studies investigating the effects of GLP1R agonists on mood disorders are limited, whereas experimental studies and systematic reviews have provided evidence supporting this association. GLP1R is widely expressed in pancreas, lungs, skin, vagus nerve, and central nervous system, implicating their potential roles in neuroprotection and psychiatric disorders [12]. Additionally, animal studies indicated neuroprotective effects as well as reversed effects on amphetamine-induced mania-like behavior [9, 10, 13]. Addressing this gap is important for developing therapeutic strategies for neuropsychiatric conditions. To bridge this knowledge gap, we aimed to assess the causal effect of GLP1R perturbation on the risk of mood disorders (MDD and BD) using Mendelian randomization (MR).

MR is a method widely applied to investigate the associations between complex traits and diseases, achieving significant success in establishing causal relationships [14]. It can avoid bias by leveraging the random allocation of genetic variants [15], acting as a ‘natural randomized controlled trial’. Recently, this method has been increasingly applied to drug target validation [16], with studies exploring additional therapeutic benefits of neurokinin 3 receptor antagonists and antidiabetic medications [15, 17, 18]. MR can test the effect of a drug target by selecting genetic proxies within the drug target region [16]. 

In this study, we tested the hypothesis that GLP1R perturbation has a protective effect on the risk of mood disorders (MDD and BD) using plasma GLP1R levels and glycated hemoglobin (HbA1c) levels. Our findings demonstrate that GLP1R perturbation is associated with a reduced risk of mood disorders. For MR using HbA1c, we also accounted for the effect of glycemic control using instrumental variables (IVs) across the genome.

## Methods

### Study design

**Fig. 1:**
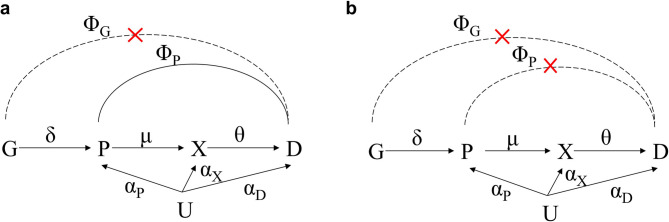
Graphs of drug target MR. **a** In the protein–drug target MR plot, exposure is the plasma protein level (P). Dashed line indicates that an assumption, Φ_G_ = 0, is needed. This model estimates the value of µθ, which is the effect of multiple pathways of the protein. **b** In biomarker-drug target MR, the effect of downstream biomarker (X) is evaluated. This model estimates the value of θ and assesses effects through downstream biomarker pathways. Assumptions Φ_G_ = 0 and Φ_P_ = 0 are needed. Abbreviations: G, genetic variant; P, protein levels; X, downstream biomarker levels; D, disease; U, common causes of both P, X, D; Φ_G_, direct effect of a genetic variant; Φ_P_, direct effect of a protein; MR, Mendelian randomization

This study utilized a two-sample MR method to evaluate the impact of GLP1R perturbation on the risk of MD, using two approaches. The first approach, referred to as protein drug target MR, used plasma levels of GLP1R as the exposure (Fig. 1a). As the exposure is closer to the genetic variants than the biomarker, the likelihood of horizontal pleiotropy is reduced [16]. This approach allows for the estimation of the effect of GLP1R protein across multiple pathways.

**Fig. 2:**
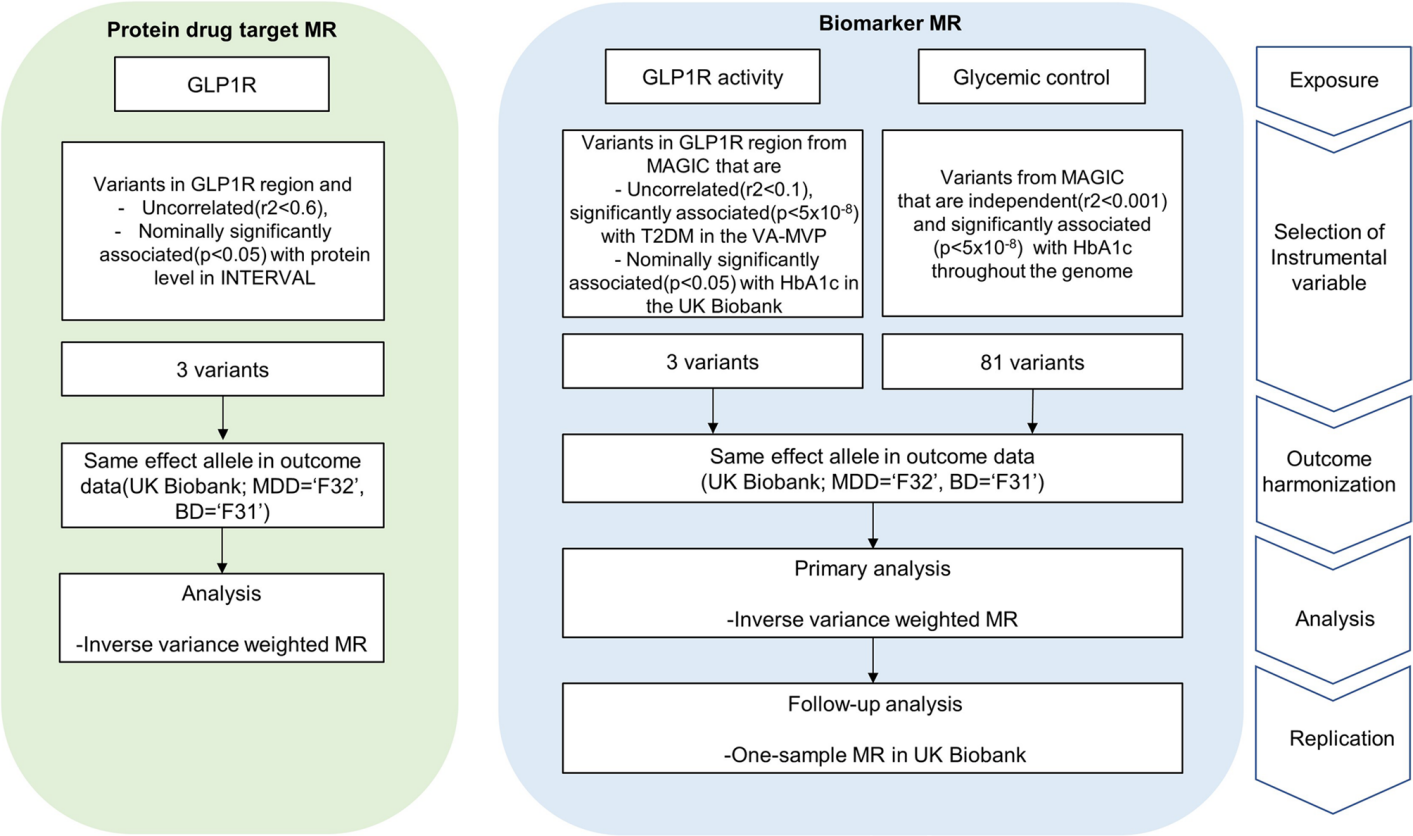
Overview of the drug target MR study between GLP1R perturbation and mood disorders

The second approach, biomarker drug target MR, focuses on the activity of GLP1R in glucose regulation using the HbA1c level (Fig. 1b). HbA1c was selected as a biomarker because GLP1R agonists are indicated for the treatment of T2DM, where HbA1c serves as a key diagnostic and monitoring marker [19]. This approach allows for the determination of the causal effect of GLP1R activity under stronger assumptions of no horizontal pleiotropy (Φ_G_ = 0, Φ_P=_ 0) [16]. We also accounted for the effect of glycemic control itself, selecting instrumental variables (IVs) across the genome (Additional Fig. 1b). This additional analysis allowed us to compare the impact of GLP1R activity with the effects of HbA1c level reduction, which helped clarify whether the psychiatric outcomes observed were more strongly influenced by GLP1R activity than by glycemic control (Fig. 2).

In an MR study, three key assumptions are required to assess the causal effect of risk factors on the outcome. First, “the genetic instruments should be associated with the risk factor (relevance).” [20] Second, “the genetic instruments should not associate with confounding (independence).” [20] Third, “the genetic instruments should influence the outcome only through the risk factor (exclusion restriction).” [20] We selected genetic instruments associated with the exposure and conducted sensitivity analyses to check for potential violations of the assumptions. In this study, we adhered to the Strengthening the Reporting of Mendelian Randomization Studies guidelines [21].

### Data sources

For the protein drug target MR, data were obtained from the INTERVAL study, which conducted a genome-wide association study (GWAS) of the concentrations of 3,622 plasma proteins from 3,301 individuals of European descent [22]. Participants were recruited into a randomized trial of blood donation frequency between mid-2012 and mid-2014, including blood donors aged 18 years and older [22]. The mean age was 43.6 years (SD 14.3) in the main subcohort (*n* = 2,481) and 44.1 years (SD 14.2) in the second subcohort (*n* = 820). The sex distribution was 51.6% male in the first subcohort and 49.5% male in the second subcohort [22]. We used summary statistics for the GLP1R plasma level in the protein drug target MR.

Summary statistics of the UK Biobank were used for BD (371 cases/360,823 controls) and MDD (776 cases/360,418 controls) defined by the International Classification of Diseases, 10th revision (ICD-10) codes ‘F31’ and ‘F32’ [23]. The UK Biobank is a large cohort of approximately 500,000 individuals across the United Kingdom, aged between 40 and 69 years at recruitment [24]. The baseline characteristics indicate a sex distribution of 273,165 females (54.4%) and 228,985 males (45.6%) [24]. In terms of self-reported ethnic background, the majority of participants identify as White (94.23%), with smaller representations from Asian, Black, and other ethnic groups [24].

For the biomarker MR, summary statistics of HbA1c were sourced from the Meta-Analyses of Glucose and Insulin-related traits Consortium (MAGIC) [25, 26]. We used European ancestry summary statistics (*n* = 128,610), which did not overlap with the UK Biobank.

### Instrumental variable selection

Proxies for the GLP1R level were chosen within the GLP1R region (± 1 MB). Variants that were uncorrelated (r^2^ < 0·6) and nominally significantly associated (*P* < 0·05) with GLP1R plasma levels in the INTERVAL study were selected. This choice was made to ensure robust MR results with multiple IVs. Uncorrelated variants were identified through linkage disequilibrium (LD) clumping using PLINK 2.0. The strengths of all IVs were calculated using F-statistics, and strong IVs (F-statistic > 10) were retained for further analysis.

To proxy GLP1R’s HbA1c level-lowering activity, we utilized the same variants identified in a study investigating the effects of GLP1R agonism on heart failure [15]. Specifically, GLP1R variants that were uncorrelated (r^2^ < 0·1) and significantly associated (*P* < 5 × 10^−8^) with T2DM in a meta-analysis [27] and nominally significantly associated (*P* < 0·05) with HbA1c in the UK Biobank were chosen [15]. To proxy glycemic control, we selected variants throughout the genome, excluding the GLP1R region. Independent variants (r^2^ < 0·001) that were genome-wide significant (*P* < 5 × 10^−8^) with HbA1c in the MAGIC meta-analysis were chosen (Fig. 2).

### Statistical analysis

Statistical analyses were performed using the MendelianRandomization and TwoSampleMR packages in R (version 4.2.2). Plots were generated using the ggplot2 package. We employed two-sample MR methods to investigate the causality of GLP1R perturbation on mood disorders, utilizing inverse-variance weighted (IVW) approaches, MR-Egger, Weighted median MR. We accounted for glycemic effects using the same approach as in a previous study. The differences between the MR estimates of GLP1R activity and glycemic control were calculated, and the standard errors were obtained using the propagation of error method [15].

### Assessment of MR assumptions and replication

The first assumption, relevance, was addressed by selecting IVs associated with the risk factors. Sensitivity analyses were performed to demonstrate the second and third assumptions. Heterogeneity was tested using Cochran’s Q test, and directional pleiotropy was evaluated using two-sample MR-Egger regression [28]. Although it is unavailable to directly test pleiotropy, we investigated associations with other phenotypes using the GWAS Catalogue (https://www.ebi.ac.uk/gwas/). For the IV that showed an association with smoking, we evaluated the mediating effect using GWAS & Sequencing Consortium of Alcohol and Nicotine use (GSCAN) summary statistics [29]. A single single nucleotide polymorphism (SNP) MR was performed by calculating the Wald ratio, which is the ratio of the coefficients of the outcome to the coefficients of exposure.

In replication, the HbA1c data (344,182 samples) in the UK Biobank were used as the exposure data [23]. We applied the two-sample MR method within the one-sample MR framework, as this approach is robust to pleiotropy and applicable to large biobank datasets [30].

## Results

### Genetic instruments of GLP1R perturbation and glycemic control

Three variants were used as proxies for GLP1R level (rs1781716, rs1699011, and rs9471005). Of the 8,328 variants from the cis-GLP1R (± 1 MB) region, four independent (r^2^ < 0·6) variants were nominally associated (*P* < 0·05) with GLP1R plasma level and three variants remained with the outcome data. As genetic instruments for GLP1R’s HbA1c level-lowering activity, three independent variants (rs10305420, rs75151020, and rs2268647) were selected. The specific summary statistics of the three IVs are provided in Additional Tables 1 and 2. Genetic proxies for glycemic control were selected from 8,230 genome-wide significant (*P* < 5 × 10^−8^) variants in the MAGIC summary statistics. Eighty-one SNPs remained after LD clumping (r^2^ < 0·001), and there were no variants in the GLP1R region (Additional Table 3).

### Effects of GLP1R level on mood disorders

**Fig. 3:**
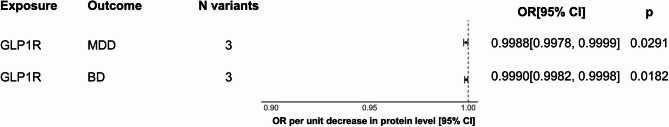
Forest plot of the protein-drug target MR for associations between GLP1R levels and mood disorders. GLP1R level, BD, and MDD data were used from INTERVAL and UK Biobank. Estimates of robust IVW MR are given as ORs with 95% CI. Abbreviations: OR, odds ratio; CI, confidence interval; MR, Mendelian randomization

Genetically proxied decreased GLP1R level was significantly associated with a decreased risk of MDD, with an odds ratio (OR) of 0·9988 (95% confidence interval [CI] = 0·9978–0·9999, *P* = 0·0291). Decreased GLP1R level was also significantly associated with a decreased risk of BD, with an OR of 0·9990 (95% CI, 0·9982–0·9998; *P* = 0·0182) (Fig. 3).

### Effects of GLP1R activity on mood disorders

**Fig. 4:**
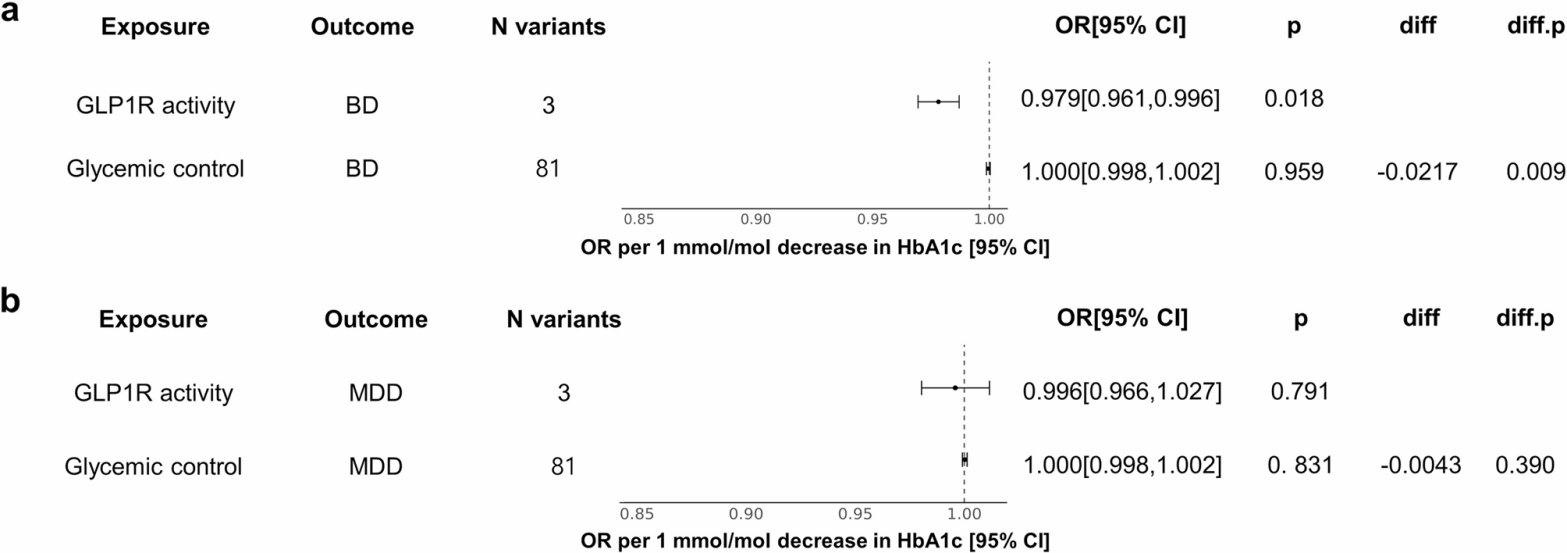
Forest plot of the biomarker-MR for associations of GLP1R activity, glycemic control and mood disorders. Association between GLP1R activity, glycemic control, and (**a**) BD and (**b**) MDD. HbA1c data were used from MAGIC and linked to BD and MDD data from UK Biobank. Estimates of robust IVW MR are given as OR with 95% CI. OR indicates the odds ratio, diff refers to the difference of estimates, and ‘diff.p’ is the p value indicating the significance of the difference. Abbreviations: OR, odds ratio; CI, confidence interval; HbA1c, glycated hemoglobin; MAGIC, Meta-Analyses of Glucose and Insulin-related traits Consortium

Figure4 shows the MR results of the HbA1c level-lowering activity of GLP1R in patients with MDD and BD. For BD, increased GLP1R activity was significantly associated with decreased risk (OR=0·979, 95% CI = 0·961–0·996, *P* =0·018) (Fig. 4a). In contrast, the genetically proxied improvement in glycemic control showed no significant association with the risk of BD (OR = 1·000, 95% CI: 0·998–1·002, *P* = 0·959). Furthermore, the difference between the estimates of GLP1R activity and glycemic control was significant in BD (*P*=0·009, Fig.4a). For MDD, no significant association was observed between increased GLP1R activity and MDD risk (OR = 0·996, 95% CI: 0·966–1·027, *P* = 0.791). Similarly, the glycemic control showed no significant association with the risk of MDD (OR= 1·000, 95% CI: 0·998–1·002, *P* = 0·831) (Fig. 4b).

### Sensitivity analysis and replication

In the sensitivity analysis, the GLP1R level IVs showed no significant heterogeneity for MDD (Q = 2·98, *P* = 0·23) or BD (Q = 3·91, *P* = 0·14). GLP1R activity also showed no evidence of heterogeneity, as the Q value was 4.53, with a p-value of 0.103 in Cochran’s Q test. The MR-Egger regression showed a non-significant intercept estimate (estimate = 0·009, *P* = 0·76). The third assumption, of no horizontal pleiotropy, was supported by scanning the GWAS catalogue (Additional Table 4). Vertical associations were also observed, such as of T2DM with rs10305420 and of body mass index with rs2268647. Although an association between smoking initiation and rs10305420 was also found, the mediation effect of rs10305420 was not significant, as indicated by single SNP MR (Wald ratio =-0·220, *P* = 0·925) (Additional Table 5).

**Fig. 5:**
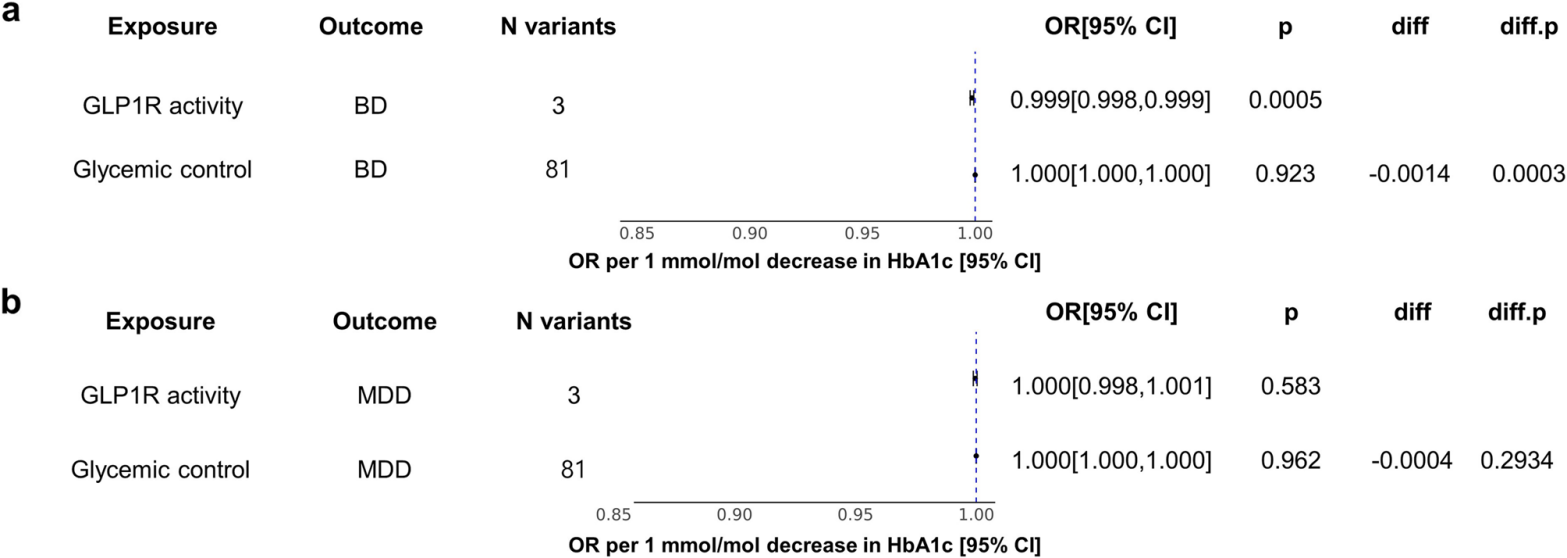
Forest plot of the one-sample MR for associations of GLP1R activity and glycemic control.Association between GLP1R activity and glycemic control with (**a**) BD and (**b**) MDD. HbA1c, BD, and MDD data were used from UK Biobank. Estimates are given as OR with 95% CI. OR indicates the odds ratio, diff refers to the difference of estimates. ‘diff.p’ is the p value that indicates the significance of the difference. Abbreviations: OR: odds ratio, CI: confidence interval, HbA1c: glycated haemoglobin

In the replicate one-sample MR, increased GLP1R activity was significantly associated with a reduced risk of BD (OR=0·999, *P* = 0·0005, Fig. 5a), whereas the association with glycemic control was not significant. The difference between the two MR estimates was significant (*P* = 0·0003) (Fig. 5a). Furthermore, weighted median MR showed significant association between increased GLP1R activity and a reduced risk of BD (OR = 0·9983, *P* = 0·037). GLP1R activity and glycemic control were not significantly associated with the risk of MDD (Fig.5b).

## Discussion

In this study, we explored the effects of GLP1R perturbation on mood disorders (MDD and BD) using genetic proxies. To examine the impact of GLP1R perturbation at two different levels, we selected variants associated with GLP1R plasma levels and HbA1c levels as proxies using an MR approach. This study showed that decreased plasma GLP1R levels were significantly associated with reduced risks of both MDD and BD. Conversely, the HbA1c level-lowering effect of GLP1R showed a protective association with BD but not with MDD. Our findings suggest that GLP1R may influence MDD and BD through distinct pathways, providing more understanding of its role. Moreover, this study provides insights into the potential protective effects of GLP1R perturbation in the risk of mood disorders.

The significant result with BD in the biomarker MR is consistent with previous findings. Rosso et al. found that patients with BD exhibit dysregulation in markers of glucose metabolism compared to healthy controls [31]. Unlike observational studies, which can be influenced by confounding factors, MR provides a causal framework that minimizes bias and reverse causation effects. Additionally, our study utilizes large-scale genetic datasets, including UK Biobank, providing increased statistical power and generalizability. Thus, our findings complement previous clinical observations and extend the evidence by providing a genetic-based causal inference of GLP1R involvement in mood disorders.

While previous drug target MR studies investigating the effects of GLP1R agonists have used glycemic traits as risk factors [15, 32], these studies were limited to assessing GLP1R's effects through metabolic pathways. In contrast, our study examines GLP1R protein levels, allowing us to capture the potential influence of all GLP1-mediated mechanisms beyond metabolic effects. However, in protein MR, a higher p-value threshold was used when selecting IVs to ensure robust MR results with multiple IVs. To our knowledge, this is the first study to apply MR to evaluate the relationship between GLP1R agonism and mood disorders, providing novel insights into the role of GLP1R signaling in two levels.

When accounting for all potential mechanisms, our results showed a significant association between decreased GLP1R levels and the reduced risk of mood disorders. Since GLP1R is a membrane protein, its blood levels do not directly reflect its activity. However, membrane protein function can be regulated through ectodomain shedding, such as canonical shedding [33]. Thus, a decrease in GLP1R protein in the blood may indicate reduced shedding, leading to a higher presence of functional GLP1R in the membrane. Our findings were consistent with previous studies that reported low serum GLP-1 levels and significant differences in GLP1R gene expression in patients with mood disorders [31, 34]. To explore potential mechanisms underlying this association, we conducted a literature review and identified several plausible explanations.

First, GLP1R agonists have been associated with the over-activation of GSK3β (glycogen synthase kinase-3β) in a depression model of chronic corticosterone (CORT) administration in mice, suggesting a potential link between GLP1R signaling and GSK3β-related pathways [35]. GSK3β polymorphisms have also been linked to reduced GM (grey matter) volume in the hippocampus and superior temporal gyrus (STG) [36], yet it remains unclear whether these structural differences precede or result from major depressive disorder (MDD), necessitating further research. Second, GLP1R agonists promoted adult neurogenesis in the dentate gyrus of the hippocampus, a process known to be reduced in both animal models of depression and human patients. [35] Given that chronic antidepressant treatments enhanced hippocampal neurogenesis, this mechanism may contribute to mood regulation; however, the direct causal link between neurogenesis reduction and depression remains uncertain. Third, GLP1 exerted anti-inflammatory effects in the central nervous system (CNS), as evidenced by its ability to significantly reduce IL-1β mRNA expression in LPS-stimulated cultures [37]. Since elevated pro-inflammatory cytokines such as IL-1β, IL-6, and TNF-α have been observed in both depressed patients and animal models, and IL-1β is known to suppress neurogenesis, GLP1R agonism may mitigate depressive symptoms through modulation of inflammatory pathways [38, 39]. Lastly, GLP1 enhanced corticotropin-releasing factor (CRF) release, activating the hypothalamic-pituitary-adrenal (HPA) axis [40]. HPA axis hyperactivation, characterized by increased cortisol secretion, elevated ACTH levels, and heightened adrenal reactivity, was observed in MDD patients [41]. Liraglutide treatment has been shown to lower ACTH levels in a depression model, suggesting a potential regulatory role of GLP1 in stress responses [35]. Given that ACTH influences HbA1c levels and a difference was observed in the biomarker MR results, this mechanism may underlie distinctions between MDD and BD. However, the causal relationship between stress response and mood disorders remains unclear, warranting further investigation.

This study has some limitations. First, we did not used directly measured GLP1R activity data(e.g., binding to ligands). Therefore, our conclusions are based on indirect assessments, and further studies with direct measurements of GLP1R function are necessary to validate our findings. Second, there was no replication of the protein drug target MR due to the limited availability of datasets. Since this study was conducted using a European cohort, further research is necessary to determine whether these results apply to other ethnic groups. Additionally, the observed effect sizes were small, which may raise concerns regarding potential false-positive findings. However, the consistency of results across multiple MR analyses (protein MR and biomarker MR) and the alignment with prior epidemiological and mechanistic studies support their biological plausibility. Lastly, the MR studies have the possibility of bias, including selection bias due to differences in survival between phenotypes [42] and misclassification bias. The UK Biobank participants are self-selected rather than randomly sampled and tend to be healthier and have higher socioeconomic status compared to the general population [43]. Therefore, our findings may not fully generalize to broader populations, and caution is needed when interpreting the results.

Our findings highlight the potential of GLP1R agonists, commonly used in metabolic disorders, as candidates for repurposing in mood disorders. However, while the MR estimates provide insights into the long-term consequences of genetically driven perturbations in GLP1R signaling, caution is required when extrapolating these findings to pharmacological effects. Nevertheless, our results support further investigation into the psychiatric implications of GLP1R modulation. Future research should incorporate clinical trial data and real-world evidence to evaluate the efficacy of GLP1R agonists in mood disorders.

## Conclusions

In conclusion, this study provides insights into the potential protective effects of GLP1R perturbation in mood disorders. By utilizing both protein and biomarker-level analyses, we were able to suggest the distinct pathways through which GLP1R may influence MDD and BD. Treatments targeting GLP1R, such as GLP1R agonists, may offer additional benefits for patients at risk of mood disorders. Further experimental studies that explore GLP1R’s molecular mechanisms are important to deepen our understanding of its psychiatric effects and to determine the therapeutic implications.

## Supplementary Information

Supplementary Material 1: Biomarker Mendelian randomization model of (a) GLP1R activity and (b) glycemic control on mood disorders.

Supplementary Material 2: Three Instrumental variables (IVs) of GLP1R activity, and their estimates for HbA1c, mood disorders in MAGIC and UK Biobank.

Supplementary Material 3: Three IVs of GLP1R level, and their estimates for GLP1R, mood disorders in INTERVAL and UK Biobank.

Supplementary Material 4: 81 IVs of glycemic control, and their estimates for HbA1c in MAGIC.

Supplementary Material 5: Scanned GWAS catalog for IVs of GLP1R level and activity

Supplementary Material 6: Additional single SNP MR analysis on the impact of smoking initiation on BD.

Supplementary Material 7: Mendelian Randomization Results for the Association Between GLP1R protein level, HbA1c, insulin, and Mood Disorders Using PGC Data.

## Data Availability

The data from the INTERVAL study is available at https://doi.org/10.1038/s41586-018-0175-2. The data from the UK biobank is available at https://www.nealelab.is/uk-biobank. The summary-level results of the meta-analysis are available on the MAGIC website (https://www.magicinvestigators.org/). GSCAN data is available at https://conservancy.umn.edu/items/91f6a003-6af2-4809-9785-53dc579dc788.

## Abbreviations

GLP1R: Glucagon-like peptide-1 receptor
MDD: Major depressive disorder
BD: Bipolar disorder
MR: Mendelian Randomization
GSCAN: GWAS& Sequencing Consortium of Alcohol and Nicotine use
GWAS: Genome-wide association study
T2DM: Type 2 diabetes mellitus
IV: Instrumental variables
LD: Linkage disequilibrium
