# Supplementary material for: Genetically proxied glucagon-like peptide-1 receptor perturbation and risk of mood disorders: a Mendelian randomization study
Source: BMC Psychiatry. 2025 Aug 6;25:768. doi: 10.1186/s12888-025-07152-0 (PMC12330103; doi:10.1186/s12888-025-07152-0)
Supplement: Supplementary file 1 — Supplementary Material 1: Biomarker Mendelian randomization model of (a) GLP1R activity and (b) glycemic control on mood disorders. [file 12888_2025_7152_MOESM1_ESM.pdf]

Additional Table 1. Three Instrumental variables (IVs) of GLP1R activity, and their estimates for HbA1c, mood disorders in MAGIC and UK Biobank. CHR: chromosome; POS: position; ID: rsid; OA: other allele; EA: effect allele; EAF: effect allele frequency; Mut: mutation type; F: F-statistics; N: number; SE: standard error; UKB: UK Biobank; BD: Bipolar disorder; MDD: major depressive disorder

								**MAGIC_HbA1c**				**UKB_HbA1c**				**UKB_BD(F31)**				**UKB_MDD(F32)**			
**CHR**	**POS**	**ID**	**OA**	**EA**	**EAF**	**Mut**	**F**	**N**	**Beta**	**SE**	**P**	**N**	**Beta**	**SE**	**P**	**N**	**Beta**	**SE**	**P**	**N**	**Beta**	**SE**	**P**
6	39016636	rs10305420	C	T	0.39	missense	10.12	128,609	-0.0038	0.0014	0.0035	344,182	-0.0509	0.0158	0.0013	361,194	$3.85e^{-5}$	$8.30e^{-5}$	0.6431	361,194	$5.86e^{-5}$	0.0001	0.632
6	39031592	rs75151020	A	C	0.09	intronic	19.36	128,610	0.0052	0.0024	0.0107	344,182	0.1189	0.0265	$7.08e^{-6}$	361,194	$1.75e^{-4}$	$1.39e^{-4}$	0.2084	361,194	$8.23e^{-5}$	0.0002	0.688
6	39043178	rs2268647	C	T	0.52	intronic	14.49	128,610	0.0030	0.0013	0.0056	344,182	0.0664	0.0154	$1.51e^{-5}$	361,194	$1.70e^{-4}$	$8.06e^{-5}$	0.0348	361,194	$6.24e^{-5}$	0.0001	0.599
