# Supplementary material for: Genetically proxied glucagon-like peptide-1 receptor perturbation and risk of mood disorders: a Mendelian randomization study
Source: BMC Psychiatry. 2025 Aug 6;25:768. doi: 10.1186/s12888-025-07152-0 (PMC12330103; doi:10.1186/s12888-025-07152-0)
Supplement: Supplementary file 2 — Supplementary Material 2: Three Instrumental variables (IVs) of GLP1R activity, and their estimates for HbA1c, mood disorders in MAGIC and UK Biobank. [file 12888_2025_7152_MOESM2_ESM.pdf]

Additional Table 3. 81 IVs of glycemic control, and their estimates for HbA1c in MAGIC. CHR: chromosome; POS: position; OA: other allele; EA: effect allele; EAF: effect allele frequency; Mut: mutation type; F: F-statistics; N: number; SE: standard error;

N	SNP	CHR	POS	EA	OA	EAF	beta	SE	P value	Sample size	Region	Gene
1	rs267738	1	150940625	T	G	0.797	0.0109	0.0016	1.14E-11	143769	exonic	CERS2
2	rs7534795	1	155275553	T	C	0.286	0.01	0.0016	2.13E-09	128610	intergenic	PKLR(dist=4324),FDPS(dist=2986)
3	rs857725	1	158607935	T	G	0.723	-0.0208	0.0014	5.43E-55	144996	exonic	SPTA1
4	rs7547793	1	203653544	A	C	0.12	-0.0118	0.0021	6.61E-09	128610	intronic	ATP2B4
5	rs340882	1	214145731	C	G	0.42	-0.0084	0.0013	1.48E-10	146806	ncRNA_intronic	PROX1-AS1
6	rs2375278	1	25529038	A	G	0.176	0.0112	0.0017	1.05E-11	140391	intergenic	MIR4425(dist=178961),SYF2(dist=19729)
7	rs1175549	1	3691727	A	C	0.786	0.0098	0.0015	7.13E-13	143770	intronic	SMIM1
8	rs560887	2	169763148	T	C	0.306	-0.0307	0.0014	5.55E-122	145580	intronic	G6PC2
9	rs13389076	2	169789512	A	G	0.034	0.0332	0.0038	3.04E-18	146806	intronic	ABCB11
10	rs17256082	2	175292364	T	C	0.628	-0.0069	0.0013	3.19E-08	144990	intronic	SCRN3
11	rs13419763	2	219134950	T	C	0.588	0.008	0.0014	5.48E-09	128610	upstream	AAMP,PNKD(dist=292)
12	rs12612492	2	24093756	T	C	0.148	0.0188	0.0019	1.88E-26	143735	intronic	ATAD2B
13	rs1367173	2	43449385	T	C	0.106	-0.0152	0.002	1.66E-14	139994	downstream	ZFP36L2(dist=156)
14	rs77503991	2	48068647	T	C	0.172	0.0088	0.0017	4.29E-08	128610	intronic	FBXO11
15	rs17037289	2	48587198	A	G	0.756	-0.0089	0.0015	2.40E-09	128610	intronic	FOXN2
16	rs10169706	2	5791194	T	C	0.04	0.026	0.0046	1.48E-08	85395.7	ncRNA_intronic	LINC01248
17	rs12491937	3	12268244	A	G	0.555	0.009	0.0013	1.42E-13	146805	intergenic	SYN2(dist=34712),PPARG(dist=60623)
18	rs11719201	3	123068744	T	C	0.182	-0.0129	0.0015	2.43E-18	146806	intronic	ADCY5
19	rs6804915	3	170627909	A	C	0.288	-0.0108	0.0014	2.76E-16	146805	intergenic	EIF5A2(dist=1483),SLC2A2(dist=86227)
20	rs4894769	3	171516306	A	T	0.413	-0.0073	0.0013	3.61E-09	135808	intronic	PLD1
21	rs13089972	3	171798694	A	T	0.584	0.0111	0.0014	1.87E-15	127524	intronic	FNDC3B
22	rs9818758	3	49382925	A	G	0.204	0.0131	0.0017	1.49E-13	143772	intergenic	USP4(dist=5439),GPX1(dist=11679)
23	rs6445541	3	52880128	T	G	0.412	0.0077	0.0014	1.14E-08	132400	intronic	STIMATE,STIMATE-MUSTN1
24	rs13134327	4	144659795	A	G	0.331	0.0144	0.0014	2.81E-26	143677	intergenic	FREM3(dist=37967),LOC105377458(dist=79152)
25	rs11729636	4	145171703	T	C	0.964	0.0263	0.0051	6.10E-10	100435	intergenic	GYPA(dist=109832),HHIP-AS1(dist=392365)
26	rs6877043	5	154048367	T	C	0.638	0.0085	0.0014	1.99E-10	128609	intergenic	MIR3141(dist=72735),LARP1(dist=14172)
27	rs1948759	5	156442657	A	G	0.166	-0.0097	0.0017	2.44E-08	146802	intergenic	TIMD4(dist=52420),HAVCR1(dist=13767)
28	rs9376090	6	135411228	T	C	0.728	0.0247	0.0014	1.90E-62	141587	intergenic	HBS1L(dist=35268),MYB(dist=91218)
29	rs6931514	6	20703952	A	G	0.736	-0.0102	0.0014	1.18E-13	146531	intronic	CDKAL1
30	rs12193223	6	24978511	C	G	0.954	0.0201	0.0034	5.87E-09	128111	intronic	RIPOR2
31	rs75580845	6	25578433	T	C	0.93	0.0203	0.0024	1.23E-20	146804	intronic	CARMIL1
32	rs1800562	6	26093141	A	G	0.046	-0.0383	0.0027	2.33E-50	146806	exonic	HFE
33	rs11758702	6	26120085	A	C	0.028	-0.0252	0.0045	6.11E-09	146322	intronic	H2BC4
34	rs13194491	6	27037080	T	C	0.07	-0.0243	0.0027	1.20E-19	126685	intergenic	LINC00240(dist=45327),H2BC11(dist=63015)
35	rs13214703	6	27941387	T	C	0.921	0.0196	0.0026	2.49E-15	143745	intergenic	OR2B6(dist=15427),OR1F12(dist=99707)

36	rs34979126	6	28449380	A	G	0.075	-0.0175	0.0026	5.60E-13	128610	intergenic	ZSCAN23(dist=38112),GPX6(dist=21693)
37	rs3129795	6	28984755	T	G	0.285	-0.0112	0.0019	2.66E-10	116632	intergenic	ZNF311(dist=11366),LOC100129636(dist=19043)
38	rs362538	6	29510630	C	G	0.106	-0.0141	0.0028	3.52E-08	118304	intergenic	LINC01015(dist=9285),UBD(dist=12662)
39	rs6929796	6	31522669	A	G	0.179	-0.0092	0.0017	3.26E-08	138645	intronic	NFKBIL1
40	rs192471087	6	32119730	C	G	0.19	0.0107	0.0019	1.37E-10	123482	intronic	PRRT1
41	rs3778321	6	7250270	A	G	0.176	-0.0106	0.0016	4.18E-11	143773	UTR3	RREB1(NM_001168344:c.*1069G>A,NM_001003698:c.*1069G>A,NM_001003700:c.*1069G>A,NM_001003699:c.*1069G>A)
42	rs7903146	10	114758349	T	C	0.307	0.0133	0.0014	1.04E-22	145580	intronic	TCF7L2
43	rs11257655	10	12307894	T	C	0.241	0.011	0.0016	1.91E-13	146802	intergenic	CDC123(dist=15306),CAMK1D(dist=83652)
44	rs2015803	10	71081399	T	C	0.265	0.0112	0.0016	2.28E-12	131081	intronic	HK1
45	rs17476364	10	71094504	T	C	0.901	0.0858	0.0023	1.33e-314	134859	intronic	HK1
46	rs11224302	11	100456604	T	C	0.096	-0.0157	0.0022	4.63E-14	132400	intergenic	CNTN5(dist=226988),ARHGAP42-AS1(dist=98288)
47	rs608793	11	118986659	T	C	0.479	0.0065	0.0013	4.55E-08	128610	intronic	C2CD2L
48	rs3842753	11	2181060	T	G	0.28	0.0075	0.0016	3.93E-08	126216	UTR3	INS(NM_001185098:c.*22C>A,NM_001185097:c.*22C>A,NM_000207:c.*22C>A,NM_001291897:c.*22C>A)
49	rs4980325	11	234451	T	G	0.532	0.0108	0.0014	4.70E-14	128610	intronic	SIRT3
50	rs11039154	11	47278502	T	C	0.277	-0.0087	0.0014	3.11E-09	146806	intronic	NR1H3
51	rs174559	11	61581656	A	G	0.285	-0.0106	0.0014	3.31E-13	146806	intronic	FADS1
52	rs10830963	11	92708710	C	G	0.714	-0.0197	0.0015	1.54E-36	145571	intronic	MTNR1B
53	rs360140	11	9776567	A	C	0.662	-0.0084	0.0013	9.62E-13	143769	ncRNA_exonic	LINC02709
54	rs10774624	12	111833788	A	G	0.525	0.0093	0.0013	4.17E-14	146805	intergenic	PHETA1(dist=26889),SH2B3(dist=9939)
55	rs17696736	12	112486818	A	G	0.559	0.0085	0.0013	4.73E-12	140004	intronic	NAA25
56	rs11066344	12	112991832	A	T	0.912	-0.0107	0.0023	2.18E-08	146801	intergenic	PTPN11(dist=44110),RPH3A(dist=21208)
57	rs117233107	12	4328521	A	G	0.02	-0.047	0.0072	8.45E-11	109012	intergenic	PARP11(dist=345907),CCND2-AS1(dist=29412)
58	rs4760682	12	48512285	A	C	0.817	0.0164	0.0018	3.20E-20	131405	exonic	PFKM
59	rs76533333	13	113352916	A	G	0.913	-0.0265	0.0025	2.81E-29	128610	intronic	ATP11A
60	rs1278769	13	113536627	A	G	0.231	-0.0091	0.0015	5.52E-12	143765	UTR3	ATP11A(NM_032189:c.*249G>A,NM_015205:c.*447G>A)
61	rs7994900	13	114553134	T	C	0.284	0.0114	0.0017	6.65E-14	106407	intronic	GAS6
62	rs1535464	14	100793431	A	G	0.212	-0.0086	0.0017	1.11E-08	128609	intronic	SLC25A47
63	rs2273475	14	65268605	A	G	0.88	-0.0128	0.0023	1.99E-09	127523	intronic	SPTB
64	rs10151436	14	73616095	A	T	0.89	0.013	0.0021	3.85E-11	128610	intronic	PSEN1
65	rs452306	15	65822777	T	C	0.627	-0.0098	0.0014	5.51E-13	128110	upstream	HACD3(dist=24)
66	rs11643024	16	11443183	A	G	0.303	0.0084	0.0015	7.98E-10	131082	intronic	RMI2
67	rs7190771	16	28590030	A	G	0.332	0.0085	0.0013	6.02E-11	146806	intronic	SGF29
68	rs11248914	16	293562	T	C	0.698	0.0114	0.0014	1.42E-14	128610	intronic	FAM234A
69	rs7198799	16	68818390	T	C	0.281	0.0083	0.0014	4.76E-09	143759	intronic	CDH1
70	rs247833	16	84581684	A	G	0.26	0.0085	0.0017	2.85E-08	128609	intergenic	MEAK7(dist=43419),COTL1(dist=17520)

71	rs837763	16	88853729	T	C	0.578	0.0176	0.0013	5.20E-38	136266	intergenic	PIEZO1(dist=2101),CDT1(dist=16468)
72	rs11656775	17	17654319	A	G	0.355	0.007	0.0014	2.50E-08	139470	intronic	RAI1
73	rs9914988	17	27183104	A	G	0.802	0.0125	0.0016	4.66E-17	144973	intronic	ERAL1
74	rs2748427	17	76121864	A	G	0.803	-0.0307	0.0022	9.82E-49	99886.8	exonic	TMC6
75	rs9909940	17	80689036	T	C	0.323	0.0322	0.0014	1.43E-116	146322	intergenic	FN3KRP(dist=3147),FN3K(dist=4455)
76	rs28671200	18	43774444	T	G	0.646	0.0086	0.0017	1.56E-08	125851	intronic	C18orf25
77	rs17533945	19	17257802	T	C	0.582	-0.0128	0.0014	1.62E-23	139480	intronic	MYO9B
78	rs10405535	19	33072085	A	G	0.29	0.0122	0.0016	6.47E-14	128609	upstream	PDCD5(dist=11)
79	rs737092	20	55990405	T	C	0.501	-0.0073	0.0013	7.57E-09	143762	intergenic	RBM38(dist=6019),CTCFL(dist=80616)
80	rs855791	22	37462936	A	G	0.4	0.0188	0.0013	1.34E-56	144995	exonic	TMPRSS6
81	rs8138197	22	43114551	A	G	0.488	-0.0073	0.0014	3.54E-08	122935	intronic	A4GALT
