# Supplementary material for: Genetically proxied glucagon-like peptide-1 receptor perturbation and risk of mood disorders: a Mendelian randomization study
Source: BMC Psychiatry. 2025 Aug 6;25:768. doi: 10.1186/s12888-025-07152-0 (PMC12330103; doi:10.1186/s12888-025-07152-0)
Supplement: Supplementary file 3 — Supplementary Material 3: Three IVs of GLP1R level, and their estimates for GLP1R, mood disorders in INTERVAL and UK Biobank. [file 12888_2025_7152_MOESM3_ESM.pdf]

Additional Table 5. Additional single SNP MR analysis on the impact of smoking initiation on BD. CHR: chromosome; POS: position; OA: other allele; EA: effect allele; AF_1000g: Allele frequency in 1000 genome data

						GSCAN_smoking int				UKB_BD				MR analysis		
**CHR**	**POS**	**RSID**	**EA**	**OA**	**AF_1000G**	**BETA**	**SE**	**P**	**N**	**BETA**	**SE**	**P**	**N**	**Wald Ratio**	**SE**	**P**
chr6	39048860	rs10305420	T	C	0.388	0.004	0.002	0.092	357,235	3.84505e-05	8.29926e-05	0.643149	361,194	0.0094011	0.0202916	0.6431
