# Supplementary material for: Genetically proxied glucagon-like peptide-1 receptor perturbation and risk of mood disorders: a Mendelian randomization study
Source: BMC Psychiatry. 2025 Aug 6;25:768. doi: 10.1186/s12888-025-07152-0 (PMC12330103; doi:10.1186/s12888-025-07152-0)
Supplement: Supplementary file 4 — Supplementary Material 4: 81 IVs of glycemic control, and their estimates for HbA1c in MAGIC. [file 12888_2025_7152_MOESM4_ESM.pdf]

Additional Table 4. Scanned GWAS catalog for IVs of GLP1R level and activity.

Risk Allele	P-value	Risk Frequency	Beta	Confidence Interval	Mapped Genes	Trait Name	Efo Traits	Accession Id	locations	Pubmed Id	author
rs10305420-C	0.000000009	0.92	0.142 unit increase	[0.093-0.191]	GLP1R	Type 2 diabetes	type 2 diabetes mellitus	GCST010556	6:39048860	32541925	Vujkovic M
rs10305420-C	3E-11	0.6161	0.0316 unit increase	[0.022-0.041]	GLP1R	Type 2 diabetes	type 2 diabetes mellitus	GCST010555	6:39048860	32541925	Vujkovic M
rs10305420-T	1E-11	0.356	0.00552 unit increase	[0.0039-0.0071]	GLP1R	Smoking initiation	smoking initiation	GCST90243985	6:39048860	36477530	Saunders GRB
rs2268647-C	0.0000003	0.499315	0.0480619 unit decrease	[0.03-0.067]	GLP1R	BMI at 1 year old	body mass index	GCST90104634	6:39075402	35315439	Helgeland
