# Supplementary material for: Genetically proxied glucagon-like peptide-1 receptor perturbation and risk of mood disorders: a Mendelian randomization study
Source: BMC Psychiatry. 2025 Aug 6;25:768. doi: 10.1186/s12888-025-07152-0 (PMC12330103; doi:10.1186/s12888-025-07152-0)
Supplement: Supplementary file 5 — Supplementary Material 5: Scanned GWAS catalog for IVs of GLP1R level and activity [file 12888_2025_7152_MOESM5_ESM.pdf]

Additional Table 2: Three IVs of GLP1R level, and their estimates for GLP1R, mood disorders in INTERVAL and UK Biobank. CHR: chromosome; POS: position; ID: rsid; OA: other allele; EA: effect allele; EAF: effect allele frequency; Mut: mutation type; F: F-statistics; N: number; SE: standard error; UKB: UK Biobank;

								**INTERVAL_GLP1R**				**UKB_BD(F31)**				**UKB_MDD(F32)**			
**CHR**	**POS**	**ID**	**OA**	**EA**	**EAF**	**Mut**	**F**	**N**	**Beta**	**SE**	**P**	**N**	**Beta**	**SE**	**P**	**N**	**Beta**	**SE**	**P**
6	38663444	rs1781716	G	C	0.08	Intronic	10.77	3,301	-0.1490	0.0454	0.0010	361,194	$-1.97e^{-4}$	0.0001	0.188	361,194	$-3.12e^{-4}$	0.0002	0.158
6	38681778	rs1699011	G	A	0.15	Upstream gene	10.58	3,301	-0.1106	0.0340	0.0011	361,194	$-1.68e^{-4}$	0.0001	0.143	361,194	$-1.69e^{-4}$	0.0002	0.317
6	39182084	rs9471005	C	T	0.03	intronic	13.84	3,301	-0.2615	0.0703	0.0002	361,194	$-6.72e^{-5}$	0.0002	0.774	361,194	$-4.39e^{-5}$	0.0003	0.899
