# Supplementary material for: Genetically proxied glucagon-like peptide-1 receptor perturbation and risk of mood disorders: a Mendelian randomization study
Source: BMC Psychiatry. 2025 Aug 6;25:768. doi: 10.1186/s12888-025-07152-0 (PMC12330103; doi:10.1186/s12888-025-07152-0)
Supplement: Supplementary file 6 — Supplementary Material 6: Additional single SNP MR analysis on the impact of smoking initiation on BD. [file 12888_2025_7152_MOESM6_ESM.pdf]

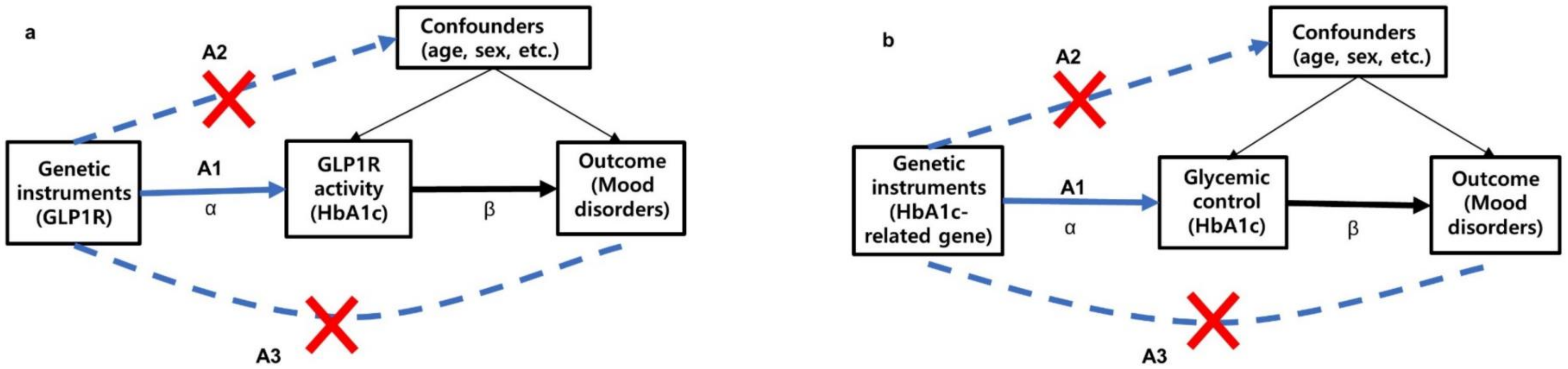

The effect of genetic variants on outcome= $\alpha \cdot \beta$

The effect of risk factor on outcome $= \frac{\alpha \cdot \beta}{\alpha} = \beta$

Assumption 1: the genetic instruments should be associated with the risk factor. “Relevance”
Assumption 2: the genetic instruments should not associate with confoundings. “Independence”
Assumption 3: the genetic instruments should influence the outcome(mood disorder) only through the risk factor. “Exclusion restriction”

Additional Figure 1. Biomarker Mendelian randomization model of (a) GLP1R activity and (b) glycemic control on mood disorders. a. In the pharmacogenetic model, genetic proxies of GLP1R activity were obtained from the GLP1R gene. b. Glycemic control was defined as the management of HbA1c level at optimum level. The effect of glycemic control was proxied by the variants associated with HbA1c throughout the genome.
