# Supplementary material for: Genetically proxied glucagon-like peptide-1 receptor perturbation and risk of mood disorders: a Mendelian randomization study
Source: BMC Psychiatry. 2025 Aug 6;25:768. doi: 10.1186/s12888-025-07152-0 (PMC12330103; doi:10.1186/s12888-025-07152-0)
Supplement: Supplementary file 7 — Supplementary Material 7: Mendelian Randomization Results for the Association Between GLP1R protein level, HbA1c, insulin, and Mood Disorders Using PGC Data. [file 12888_2025_7152_MOESM7_ESM.pdf]

Additional Table 6. Mendelian Randomization Results for the Association Between GLP1R protein level, HbA1c, insulin, and Mood Disorders Using PGC Data

EXPOSURE	OUTCOME	Values.Method	Values.Estimate	Values.Std Error	Values.P-value
GLP1R level	MDD	Simple median	0.2595	0.1078	0.0161
GLP1R level	MDD	Weighted median	0.2231	0.0966	0.0209
GLP1R level	MDD	Penalized weighted median	0.2846	0.1270	0.0250
GLP1R level	MDD	IVW	0.1536	0.0639	0.0163
GLP1R level	MDD	Penalized IVW	0.2515	0.0773	0.0011
GLP1R level	MDD	Robust IVW	0.1571	0.0471	0.0009
GLP1R level	MDD	Penalized robust IVW	0.2543	0.0372	0.0000
GLP1R level	MDD	MR-Egger	-0.2653	0.2818	0.3463
GLP1R level	MDD	(intercept)	0.0666	0.0424	0.1158
GLP1R level	MDD	Penalized MR-Egger	-0.2653	0.2818	0.3463
GLP1R level	MDD	(intercept)	0.0666	0.0424	0.1158
GLP1R level	BD	Simple median	0.0967	0.0914	0.2899
GLP1R level	BD	Weighted median	0.0943	0.0907	0.2988
GLP1R level	BD	Penalized weighted median	0.0943	0.0907	0.2988
GLP1R level	BD	IVW	0.1051	0.0689	0.1270
GLP1R level	BD	Penalized IVW	0.1051	0.0689	0.1270
GLP1R level	BD	Robust IVW	0.1045	0.0413	0.0114
GLP1R level	BD	Penalized robust IVW	0.1045	0.0413	0.0114
GLP1R level	BD	MR-Egger	0.0195	0.2183	0.9287
GLP1R level	BD	(intercept)	0.0134	0.0324	0.6797
GLP1R level	BD	Penalized MR-Egger	0.0195	0.2183	0.9287
GLP1R level	BD	(intercept)	0.0134	0.0324	0.6797
HbA1c	MDD	Simple median	-0.9427	2.5181	0.7081
HbA1c	MDD	Weighted median	-1.5862	2.3663	0.5027
HbA1c	MDD	Penalized weighted median	-2.2239	8.4354	0.7921
HbA1c	MDD	IVW	-0.5741	1.6059	0.7207
HbA1c	MDD	Penalized IVW	-0.5741	1.6059	0.7207
HbA1c	MDD	Robust IVW	-0.6222	1.0176	0.5409
HbA1c	MDD	Penalized robust IVW	-0.6222	1.0176	0.5409
HbA1c	MDD	MR-Egger	-11.9316	15.5955	0.4442
HbA1c	MDD	(intercept)	0.0431	0.0581	0.4575
HbA1c	MDD	Penalized MR-Egger	-11.9316	15.5955	0.4442
HbA1c	MDD	(intercept)	0.0431	0.0581	0.4575
HbA1c	BD	Simple median	-0.2369	2.0956	0.9100
HbA1c	BD	Weighted median	-0.3036	1.9930	0.8789
HbA1c	BD	Penalized weighted median	0.0265	2.1766	0.9903
HbA1c	BD	IVW	-1.6302	1.6716	0.3295
HbA1c	BD	Penalized IVW	-1.6302	1.6716	0.3295
HbA1c	BD	Robust IVW	-1.5449	1.0671	0.1477
HbA1c	BD	Penalized robust IVW	-1.5449	1.0671	0.1477
HbA1c	BD	MR-Egger	14.7360	8.4971	0.0829
HbA1c	BD	(intercept)	-0.0621	0.0316	0.0495
HbA1c	BD	Penalized MR-Egger	14.7360	8.4971	0.0829
HbA1c	BD	(intercept)	-0.0621	0.0316	0.0495
Insulin	MDD	Simple median	0.4581	1.8712	0.8066
Insulin	MDD	Weighted median	1.4416	1.8269	0.4301
Insulin	MDD	Penalized weighted median	1.4416	1.8269	0.4301
Insulin	MDD	IVW	1.1856	1.1429	0.2996
Insulin	MDD	Penalized IVW	1.1856	1.1429	0.2996
Insulin	MDD	Robust IVW	1.1911	0.4330	0.0059
Insulin	MDD	Penalized robust IVW	1.1911	0.4330	0.0059
Insulin	MDD	MR-Egger	2.8180	3.5976	0.4334
Insulin	MDD	(intercept)	-0.0107	0.0188	0.5689
Insulin	MDD	Penalized MR-Egger	2.8180	3.5976	0.4334
Insulin	MDD	(intercept)	-0.0107	0.0188	0.5689
Insulin	BD	Simple median	-0.8318	2.1521	0.6991
Insulin	BD	Weighted median	-2.1138	1.8116	0.2433
Insulin	BD	Penalized weighted median	-2.1138	1.8116	0.2433
Insulin	BD	IVW	-2.1632	1.1979	0.0709
Insulin	BD	Penalized IVW	-2.1632	1.1979	0.0709
Insulin	BD	Robust IVW	-0.8289	0.0148	0.0000
Insulin	BD	Penalized robust IVW	-0.8289	0.0158	0.0000
Insulin	BD	MR-Egger	-1.3512	2.6530	0.6105
Insulin	BD	(intercept)	-0.0053	0.0138	0.7002
Insulin	BD	Penalized MR-Egger	-1.3512	2.6530	0.6105
Insulin	BD	(intercept)	-0.0053	0.0138	0.7002
